# Self-assembled *α*-Tocopherol Transfer Protein Nanoparticles Promote Vitamin E Delivery Across an Endothelial Barrier

**DOI:** 10.1038/s41598-017-05148-9

**Published:** 2017-07-10

**Authors:** Walter Aeschimann, Stefanie Staats, Stephan Kammer, Natacha Olieric, Jean-Marc Jeckelmann, Dimitrios Fotiadis, Thomas Netscher, Gerald Rimbach, Michele Cascella, Achim Stocker

**Affiliations:** 1 University of Bern, Department of Chemistry and Biochemistry, Bern, Switzerland; 2 University of Kiel, Institute of Human Nutrition and Food Science, Kiel, Germany; 3 Paul Scherrer Institut, Villigen, Switzerland; 4 University of Bern, Institute of Biochemistry and Molecular Medicine, Bern, Switzerland; 5 DSM Nutritional Products Ltd., Basel, Switzerland; 6 University of Oslo, Department of Chemistry and Centre for Theoretical and Computational Chemistry (CTCC), Oslo, Norway

## Abstract

Vitamin E is one of the most important natural antioxidants, protecting polyunsaturated fatty acids in the membranes of cells. Among different chemical isoforms assimilated from dietary regimes, RRR-*α*-tocopherol is the only one retained in higher animals. This is possible thanks to *α*-Tocopherol Transfer Protein (*α*-TTP), which extracts *α*-tocopherol from endosomal compartments in liver cells, facilitating its distribution into the body. Here we show that, upon binding to its substrate, *α*-TTP acquires tendency to aggregation into thermodynamically stable high molecular weight oligomers. Determination of the structure of such aggregates by X-ray crystallography revealed a spheroidal particle formed by 24 protein monomers. Oligomerization is triggered by refolding of the N-terminus. Experiments with cultured cell monolayers demonstrate that the same oligomers are efficiently transported through an endothelial barrier (HUVEC) and not through an epithelial one (Caco-2). Discovery of a human endogenous transport protein with intrinsic capability of crossing endothelial tissues opens to new ways of drug delivery into the brain or other tissues protected by endothelial barriers.

## Introduction

The generic term vitamin E describes a group of eight plant-derived chromanols identified in 1922 by Evans and Bishop as an essential dietary fertility factor in rats^1, 2^. Out of all eight isoforms of vitamin E, which pass through the intestinal epithelium to a similar extend^3^, higher animals typically retain only RRR-*α-*tocopherol (*α*-Tol hereafter)^4–6^. *α*-Tol is a potent lipid-soluble antioxidant capable of protecting biological membranes from peroxyl radical damage and of promoting the repair of mechanically injured cells^7–10^. Retention of *α*-Tol in animals requires *α*-tocopherol transfer protein (*α*-TTP) in the liver, a cytosolic transporter of 32 kDa weight. *α*-TTP belongs to the Sec-14 like family, a protein group involved in secretion and lipid transfer^11–15^. *α*-TTP recognises and selectively extracts *α*-Tol from the endosomal fraction of the hepatocytes allowing for its transfer into the inner leaflet of the plasma membrane and eventually across the plasma membrane into the blood-stream^16–21^. Failure of this transfer leads to vitamin E deficiency, a malfunction that is associated with developmental failure in the vertebrate embryo and also in the early central nervous system^22, 23^. In developed organisms, low levels of *α*-Tol in peripheral nerves are associated with a neurological phenotype termed Ataxia with Vitamin E Deficiency^24–27^. Evidences from both human and animal studies suggest *α*-TTP as an essential protein factor for the female reproductive system^28, 29^. Detection of *α*-TTP in rodent uterus, placenta and in human yolk sac indicates that this protein may have a more general role than only delivery of *α*-Tol into the blood stream^30, 31^.

The detailed molecular mechanisms regulating *α*-Tol homoeostasis in the body are not fully understood. A hypothetical pathway from the liver to the target cell must involve the capturing of *α*-Tol from the late endosome outer leaflet and its transportation to the cytosolic facing leaflet of the plasma membrane^18, 32^. In recent times, two independent research groups suggested that transfer of *α*-Tol to the plasma membrane is coupled to the extraction of phosphatidylinositolphosphates (PIPs) from the same membrane by *α*-TTP^16, 33^. Incorporation of *α*-Tol in the outer leaflet may occur by flippase activity^34^ or by participation of the ATP-binding cassette transporter ABCA1^17, 35^. It was noticed that, after secretion, major fractions of *α*-Tol appear in nascent very low density lipoprotein in the perisinusoidal space (space of Disse)^36^. Incorporation of *α*-Tol into recipient cells is still not well understood. In particular, *α*-Tol is essential to the reproductive apparatus and the central nervous system, which are both protected by endothelial barriers. Appropriate mechanisms for crossing these barriers have not been described so far^28, 37^. Here we show that, upon binding to *α*-Tol, *α*-TTP is formed acquiring tendency to aggregation into high molecular weight oligomers. X-ray diffraction reveals a spheroidal nanoparticle formed by 24 *α*-TTP protomers of ≈17 nm diameter. We verify that such particles are efficiently and selectively transported through a model endothelium tissue from human umbilical vein, with a 28-fold increased flux with respect to paracellular flux. As aggregation is facilitated by the presence of negatively charged lipids, the assembly of the nanoparticle may occur *in vivo* at the plasma membrane after interaction with PIPs, consistently with previous findings^16^.

## Results

### *α*-Tol-induced formation of *α*-TTP nano-cages

**Figure 1:**
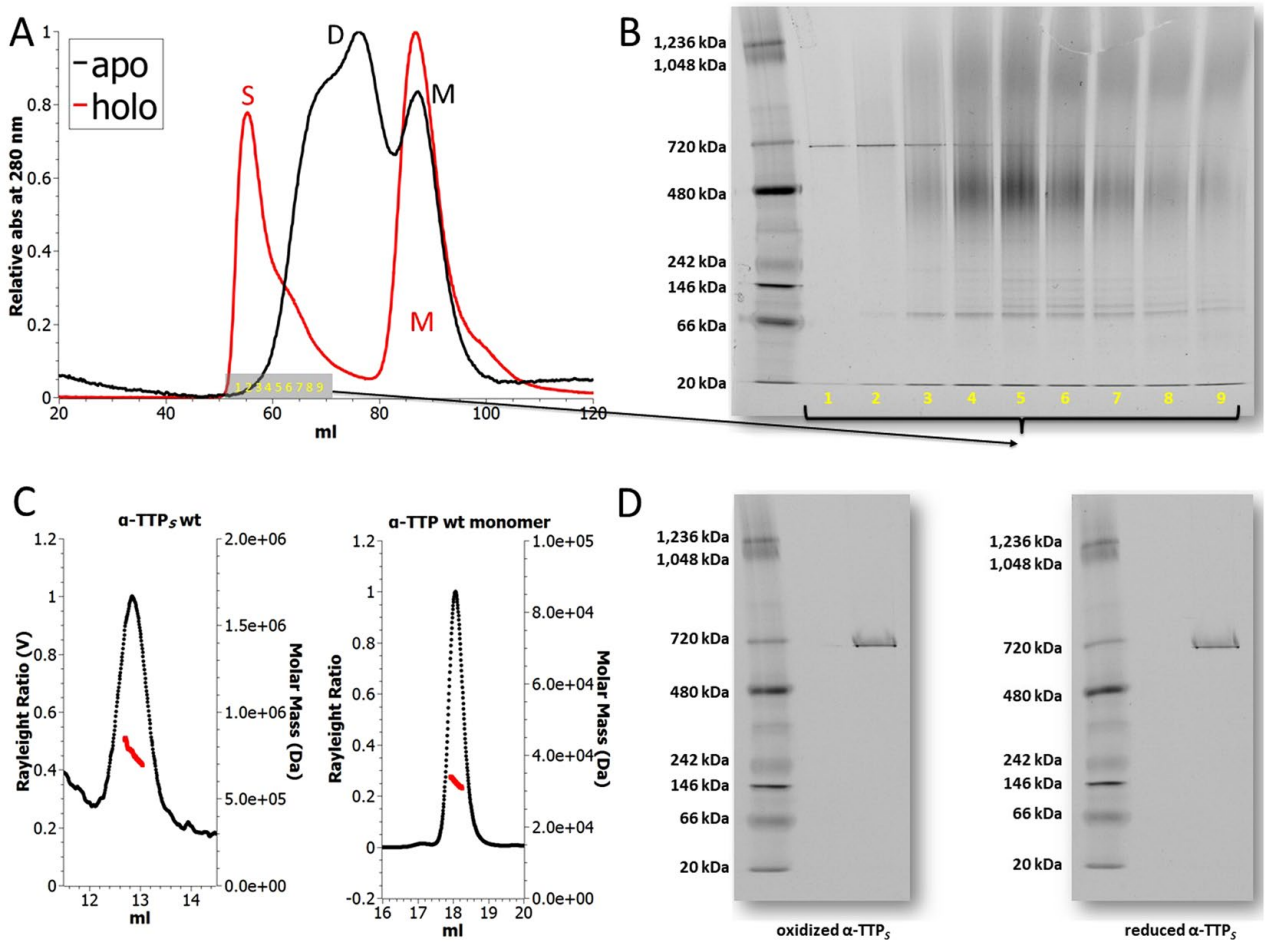
Formation of *α*-TTP homo-oligomers in presence of *α*-Tol. (**A**) SEC traces of apo-*α*-TTP (black trace) and *α*-TTP (red trace) in a preparative SEC setup. (**B**) Native PAGE of peak fraction S from preparative SEC. (**C**) SEC-MALS profiles of re-chromatographed peak fraction S and of monomeric *α*-TTP; the derived molar masses were 0.76 MDa ± 2.66% and 3.24 kDa ± 1.14% respectively. (**D**) Native PAGE of re-chromatographed peak fraction S after overnight incubation at room temperature in 10% v/v DMSO or reduction in 50 mM DTT. Disassembly of *α*-TTP_*S*_ into lower oligomers was not observed in either case.

Freshly prepared samples of ligand-free *α*-TTP (apo-*α*-TTP) dialysed in solutions of anionic detergent in the presence or absence of *α*-Tol were subjected to preparative size exclusion chromatography (SEC). In the presence of *α*-Tol two major peaks were obtained representing the ligand complexes of monomeric *α*-TTP and of its high molecular weight aggregates (Fig. 1A). The content of the high molecular weight SEC peak fraction S (fractions 1–9) was resolved by native gel electrophoresis (PAGE) revealing the presence of a sharp band of oligomeric *α*-TTP (*α*-TTP_*S*_; apparent mass 720 kDa) and of lower molecular weight forms of oligomeric *α*-TTP (Fig. 1B). Re-chromatography of the SEC peak fraction S by analytical SEC yielded a major peak of high molecular weight *α*-TTP oligomers and a minor peak representing monomeric *α*-TTP (Fig. S1). The re-appearance of monomeric *α*-TTP in the re-chromatography step indicated reversible equilibration between the states. Imaging of high molecular weight *α*-TTP oligomers by transmission electron micrography (TEM) displayed spheroidal particles with a broad Gaussian distribution of the diameter centred around 17.5 ± 4 nm (Fig. S1). Dynamic light scattering measurements in solution consistently reported a diameter of 17.6 ± 4 nm. Temperature-dependent circular dichroism spectroscopy of apo-*α*-TTP, *α*-TTP and *α*-TTP_*S*_ reported an increase in the respective melting temperatures (*T*_*m*_) from 51 °C to 68 °C and >90 °C, indicating that the monomeric ligand complex of *α*-TTP is more stable than its apo-form and further stabilised by self-aggregation (Fig. S1F). The fraction of oligomeric *α*-TTP_*S*_ that produced a sharp band in native PAGE was separated from the *α*-TTP oligomeric states of lower molecular weight by analytic SEC. The separated fraction of oligomeric *α*-TTP_*S*_ was analysed for integrity and size by SEC coupled to multi-angle light scattering (MALS-SEC) revealing a mass of 760 kDa ± 2.6% (Fig. 1C). Incubation of fractions of oligomeric *α*-TTP overnight at room temperature under mild oxidising conditions (10% v/v DMSO)^38^ or under reducing conditions (50 mM DTT) respectively did not evidence any re-equilibration into other states in native PAGE (Fig. 1D). It was concluded that the 760 kDa peak fraction *α*-TTP_*S*_ represents a stable, kinetically trapped form of oligomeric *α*-TTP_*S*_. The oligomeric *α*-TTP_*S*_ forms of the 760 kDa peak fraction were exclusively observed in the *α*-Tol bound state. In the absence of *α*-Tol, apo-*α*-TTP was detected mostly in monomeric or homo-dimeric states both in preparative and in analytical SEC (Fig. 1A, Fig. S1).

### Crystal structure of tetracosameric *α*-TTP_*S*_

**Figure 2:**
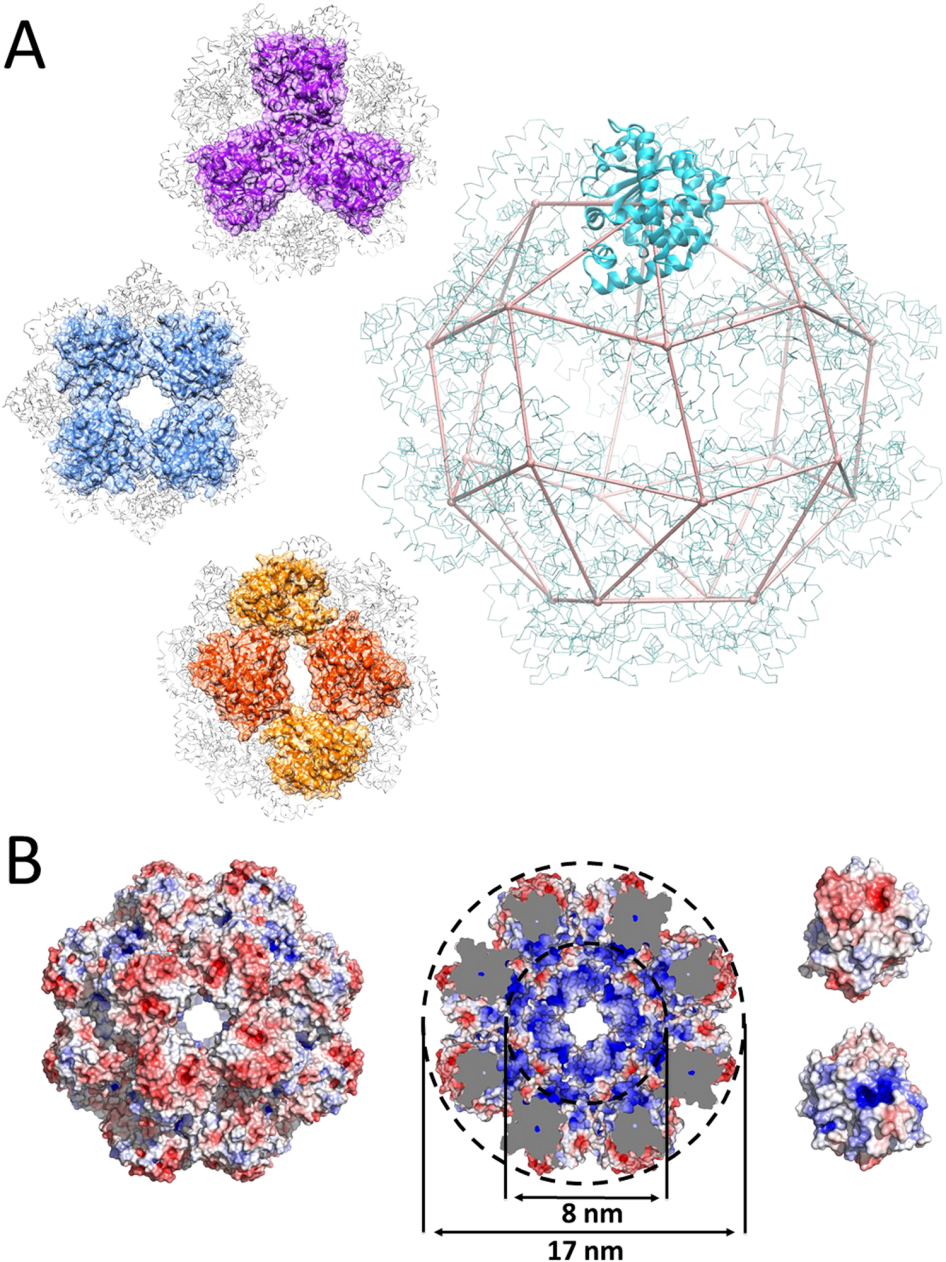
Atomic models of the tetracosameric assembly of *α*-TTP_*S*_. (**A**) View of the atomic model along the three symmetry axes; four-fold in blue (channel width ≈21 Å), three-fold in purple and two-fold in orange. In the centre a geometric representation of the twisted cantellated cube (TCC) with a ribbon cartoon of a monomer sitting on a node is shown. (**B**) Electrostatic maps of the tetracosameric assembly and of *α*-TTP; the outer sphere surface of the tetracosamer is mainly negatively charged (left) whereas its interior is mainly positively charged (middle); electrostatic maps of the external and internal faces of *α*-TTP (right).

**Figure 3:**
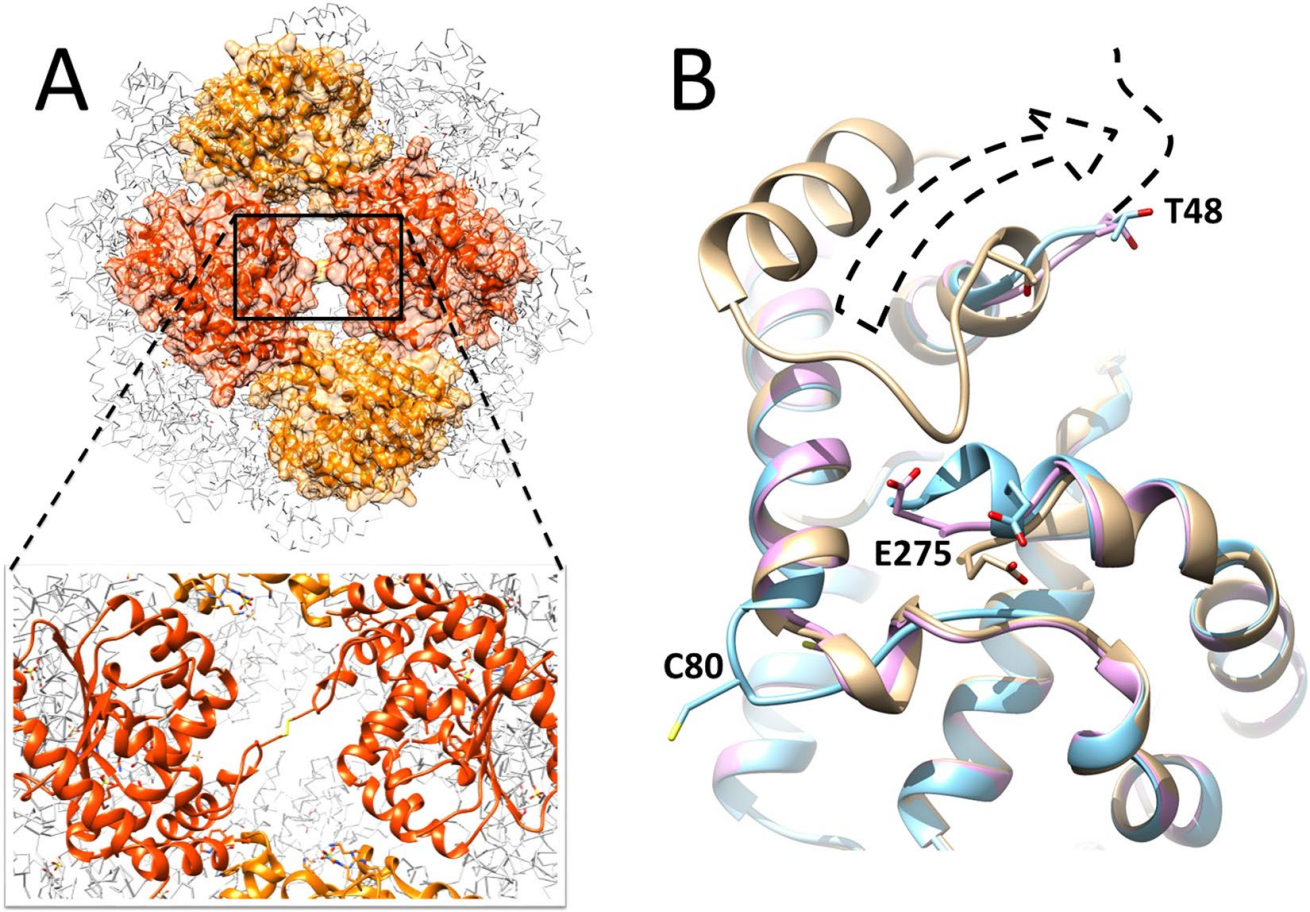
(**A**) Cysteine bond formation between two adjacent C80 across the rhombohedral channel along the two-fold symmetry axis in the oxidised form of *α*-TTP_*S*_. (**B**) Comparison of the structures in the proximity of the C-terminus. Monomeric *α*-TTP (tan); *α*-TTP_*S*_ reduced state (pink); *α*-TTP_*S*_ oxidised state (cyan). The dashed arrow highlights the large conformational change at the N-terminus (aa’s 1–47) required to expose the trimeric interface.

*α*-TTP_*S*_ crystallised exclusively when starting from monodisperse solutions of monomeric *α*-TTP. *α*-TTP_*S*_ can exist in different oxidation states; we obtained crystals in both the fully reduced and the fully oxidised states. X-ray diffraction patterns indicated that both crystals belong to the space group I432, with essentially identical unit-cell parameters a = 168.18 Å (reduced) and a = 168.26 Å (oxidised). The crystal structures were solved by molecular replacement method using the atomic model of monomeric *α*-TTP (PDB code 1OIP^39^) and refined at 2.40 Å resolution (reduced form; R_*work*_:R_*free*_ 18.2%:21.5%) and at 2.42 Å resolution (oxidized form; R_*work*_:R_*free*_ 19.1%:21.2%) respectively (see Table 1). In both cases, the asymmetric unit consists of a single *α*-TTP molecule (residues 48–278) with one *α*-Tol ligand bound to it. *α*-TTP_*S*_ is formed by 24 protomers, for a molecular mass of 0.76 MDa, assembled into a spheroidal shell reminiscent of a viral capsid (Fig. 2). Inspection of the density map of the *α*-TTP_*S*_ model revealed well defined density for *α*-Tol indicating that the protomers in the particle are bound to the ligand (Fig. S3). The X-ray structural model of *α*-TTP_*S*_, for both redox states, has an external diameter of 16.8 nm consistent in size with measurements on *α*-TTP_*S*_ particles from soluble preparations (Fig. S1). The structure is further characterised by an apparently hollow cavity of 8.1 nm diameter. Analysis of the electrostatic map at the solvent accessible surface indicates a positive potential in the cavity, corresponding to the localisation of the basic amino acids responsible for binding of PIPs as described by Arai and coworkers^16^. The chiral *α*-TTP_*S*_ assembly takes the topology of a twisted cantellated cube (point symmetry group *O*, Schonflies notation) taking the centres of each protein monomer as vertexes, and connecting them through protein-protein contact interfaces. According to its point group, *α*-TTP_*S*_ symmetry operations are proper rotations only around three *C*_4_, four *C*_3_ and six *C*_2_ axes (Fig. 2). Each *α*-TTP monomer is in contact with four first neighbours. With two of such neighbours, it forms one of the eight trimeric interfaces crossed by the *C*_3_ symmetry axes, and with the second two neighbours it is involved in building up one of the six tetrameric interfaces crossed by one of the *C*_4_ axes. The tetrameric unit is completed by a second neighbour unit, which is anyway not in direct contact. Each tetrameric unit is characterised by an open channel of ≈21 Å width (Fig. 2). Twelve rhomboidal faces complete the nano cage, each crossed by one of the *C*_2_ symmetry axes. The two asymmetric edges of such interfaces correspond to those of the trimeric and tetrameric assemblies. Each monomer is involved in two of these interfaces. The oxidised form of *α*-TTP_*S*_ presents here one disulphide bridge crosslinking C80 of two *α*-TTP units through the rhombohedral channel (Fig. 3A). The oxidised *α*-TTP_*S*_ particle thus contains a total of 12 S-S bridges covalently binding all trimeric subunits. Oxidation is accompanied by local unwinding of the helical segment (aa’s 65–79). It also induces structuring of the neighbouring C-terminus (aa’s 275–278) into a regular *α*-helical motif (Fig. 3B). No other significant structural differences are observed between the reduced and oxidised forms of *α*-TTP.

**Table 1:** Data collection and refinement statistics.

PDB ID	*α*-TTP_*S*_ (reduced state)	*α*-TTP_*S*_ (oxidized state)
	5MUE	5MUG
Crystal parameters	I432	I432
Cell dimensions *a, b, c* (Å)	168.18, 168.18, 168.18	168.26, 168.26, 168.26
*α,β,γ* (^◦^)	90, 90, 90	90, 90, 90
Data Collection		
Wavelength, Å	0.9998	1.0079
Resolution (Å) (outer shell)	48.55–2.40 (2.54–2.40)	48.57–2.42 (2.57–2.42)
No. observations	174351	139722
No. unique reflections	16228	15731
Mean redundancy	9.31 (9.53)	11.56 (11.31)
Completeness (%)	99.7 (99.1)	98.6 (95.9)
*Rmeas* %	9.50 (162.1)	9.60 (134.3)
I/*σ*(I)	21.80 (1.54)	14.00 (1.41)
CC (1/2)	99.9 (59.9)	99.8 (52.9)
Refinement Resolution range (Å)	39.64–2.40	48.57–2.42
No. reflections working set	16229	15736
No. reflections test set	809	786
*Rwork/Rfree*	18.2/21.5	19.1/21.2
rms bonds, (Å)	0.002	0.003
rms angels, (^◦^)	0.617	0.680
Residues included	48–275	48–278
Ramachandran statistic Generously allowed, %	100	100
Not allowed, %	0	0

### Protein-protein interfaces

**Figure 4:**
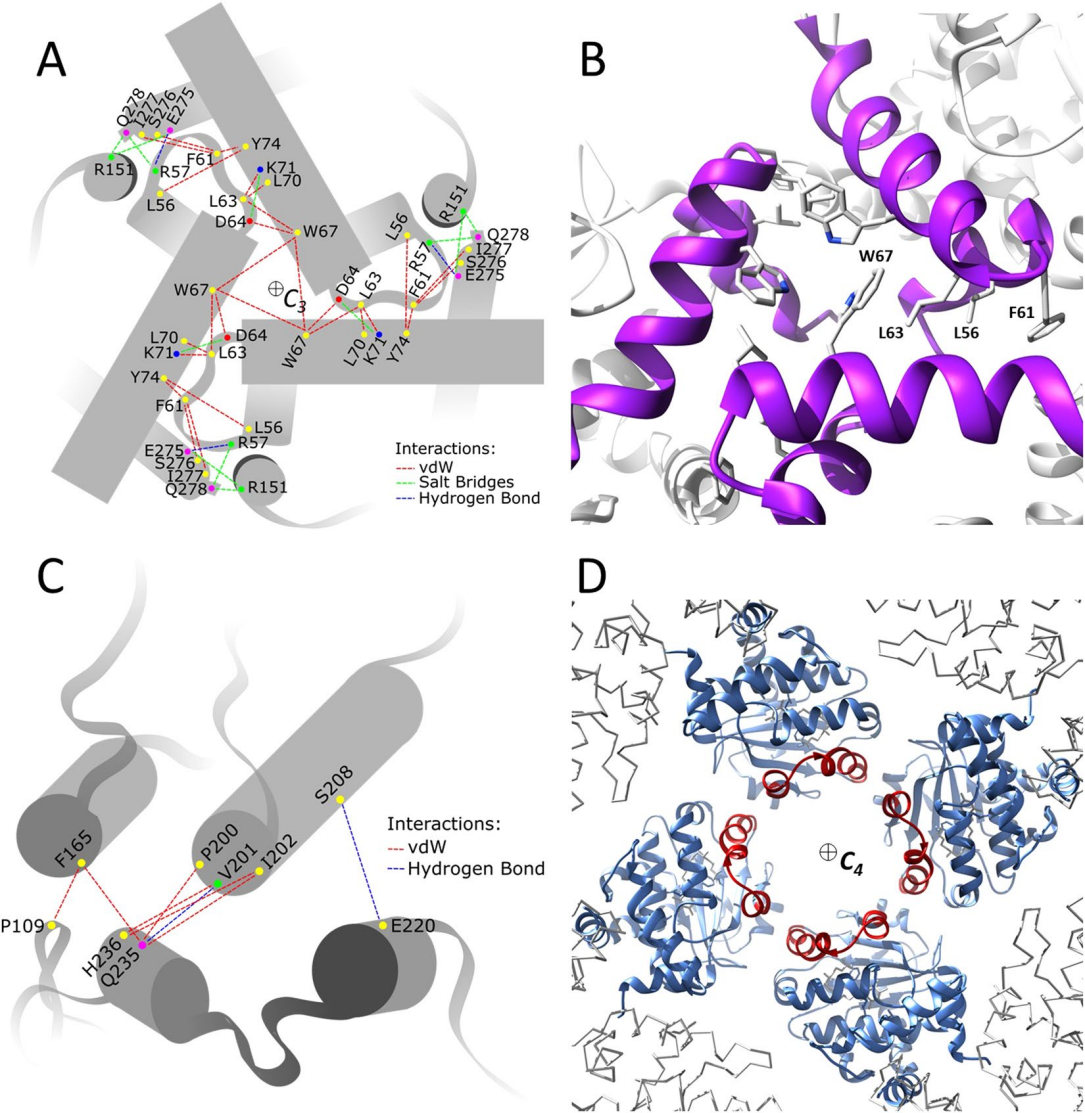
Schematic view of protein-protein interactions on the three-fold axis (**A**) and on the four-fold axis (**C**) (see Table 2 for details). (**B**) Close up view of the region around the three-fold axis. W67 forms a T-shaped van der Waals interaction with the adjacent homologs; this is the only point where a monomer interacts with two neighbours. The hydrophobic contact area of residues W67, L63, F61 and L56 defines a “hot-spot” motif in which binding energy is largely concentrated. (**D**) View from the interior along the *C*_4_ axis; the mobile gate moieties are coloured red to emphasise the steric hindrance is interfering with the release of *α*-Tol.

The highly packed trimeric interface is constituted by several protein-protein contacts. Each unit interacts with the following one through the helical segment 49–56, the 57–64 loop, and the first turn of the helical segment 65–79, as well as with residue R151. The partner protein interacts with the amino acids 67–74 in the helical segment 65–79, and with the C-terminal residues (aa’s 275 to 278) (see Fig. 4 and Table 2 for details). The trimeric interface is characterised by hydrophobic packing and further stabilised by salt bridges. These electrostatic interactions are localised mostly at the exterior of the interface, with residues R57 and R151 on one protein interacting with the C-terminus of the facing unit. Residues D64 and K71 constitute one additional salt bridge (3.87 Å), localised closer to the core of the interface. At the very centre, the three W67 residues interact with each other in T-shape by van der Waals stacking. To our observation, this is the only contact point involving more than two proteins in the whole *α*-TTP_*S*_. W67 together with L63, F61 and L56 constitute a classical hot spot that accounts for roughly three quarters (77%) of the interface’s overall binding free energy^40, 41^ (Fig. 4A). The protein-protein interface at the four-fold symmetry shows a smaller area of contacts than the three-fold one. We observe only one moderately strong H-bond bridge (3.13 Å) between the backbone of V201 and the side-chain of Q235 of the adjacent subunit. Hydrophobic interactions involve F165, P200, V201 and I202 on one protomer, and P109, Q235 and H236 on the neighbouring one. Finally, a weak H-bond (4.04 Å may form between the side-chains of S208 and E220. The most prominent feature of the tetrameric interface is its intrinsic steric properties preventing the opening of the mobile gate, thus hindering substrate release (Fig. 4D).

**Table 2:** Summary of protein-protein interactions contributing to the stability of *α*-TTP_*S*_.

2-fold symmetry axis Interface		
**Δ** ^**i**^ **G [kcal mol** ^**−1**^ **]**	**Interface area [Å** ^**2**^ **]**	**CSS**
−4.80	72.10	0.091
**Residue 1**	**Residue 2**	**Interaction**
C80	C80	Cysteine
**3-fold symmetry axis Interface**		
**Δ** ^**i**^ **G [kcal mol** ^**−1**^ **]**	**Interface area [Å** ^**2**^ **]**	**CSS**
−4.70	447.50	0.217
**Residue 1**	**Residue 2**	**Interaction**
R151	E275, Q278	Salt Bridge
R57	Q278	Salt Bridge
R57	E275	Hydrogen Bond
F61	Y74, I277, S276	vdW
L56	Y74	vdW
D64	K71	Salt Bridge
L63	K71, L70, W67	vdW
W67	W67, D64, L63	vdW
**4-fold symmetry axis Interface**		
**Δ** ^**i**^ **G [kcal mol** ^**−1**^ **]**	**Interface area [Å** ^**2**^ **]**	**CSS**
−3.50	243.6	0.071
**Residue 1**	**Residue 2**	**Interaction**
P109	F165	vdW
H236	V201, I202	vdW
Q235	F165, P200, I202	vdW
Q235	V201	Hydrogen Bond
E220	S208	Hydrogen Bond

The PDBePISA^84^ web-based tool was used to evaluate protein-protein interactions. **Δ** ^**i**^ **G** indicates the solvation free energy gain upon formation of the interface, in kcal/M. The value is calculated as difference in total solvation energies of isolated and interfacing structures. Negative Δ^i^G corresponds to hydrophobic interfaces, or positive protein affinity. This value does not include the effect of satisfied hydrogen bonds and salt bridges across the interface. **Interface area** in Å^2^, calculated as difference in total accessible surface areas of isolated and interfacing structures divided by two. **CSS** stands for the Complexation Significance Score, which indicates how significant for assembly formation the interface is. The score is defined as a maximal fraction of the total free energy of binding that belongs to the interface in stable assemblies.

### Transcytosis of *α*-TTP_*S*_

**Figure 5:**
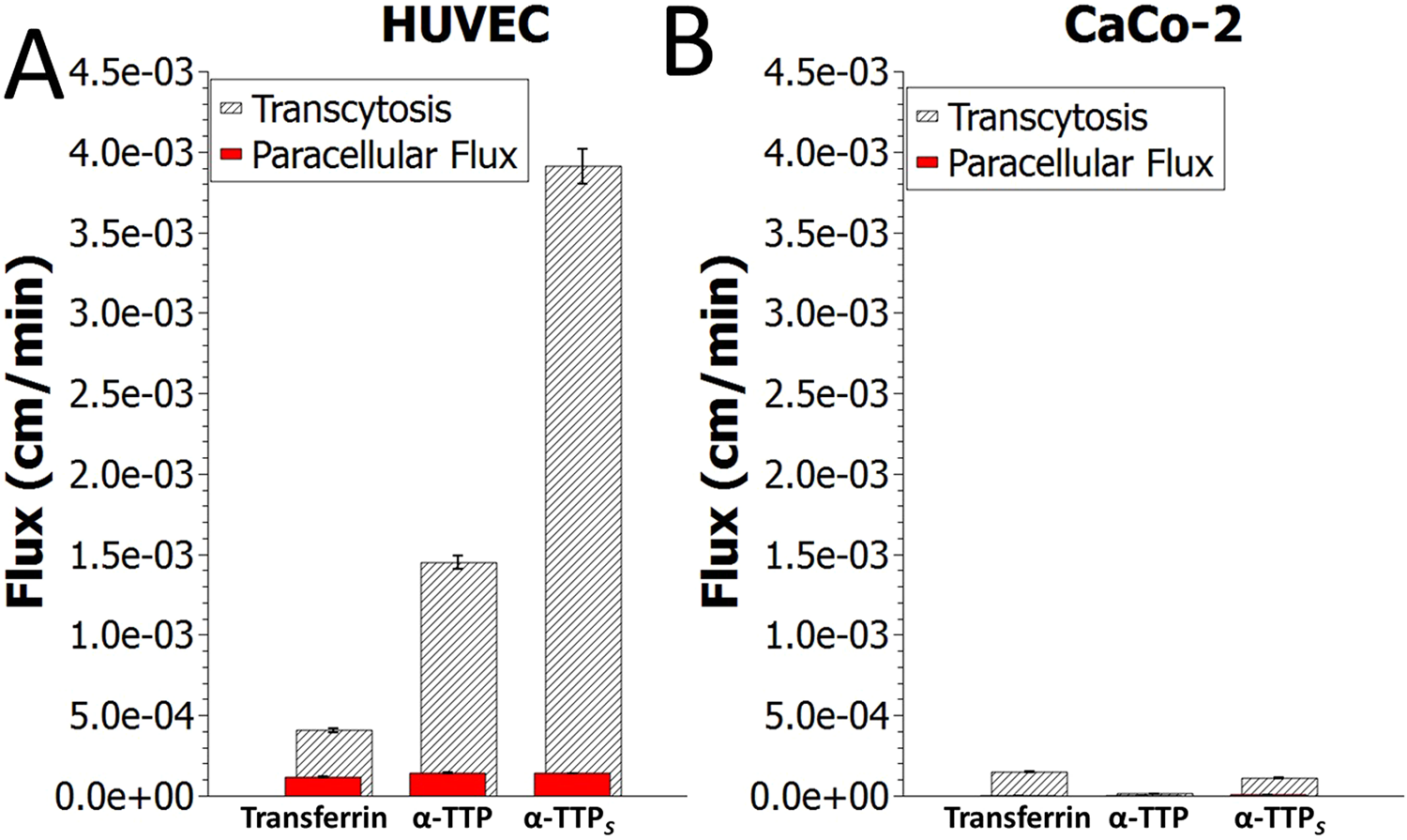
(**A**) Flux-rates of transcytosis across a human umbilical vein endothelial cell (HUVEC) monolayer. (**B**) Flux-rates of transcytosis across a heterogeneous human epithelial colorectal adenocarcinoma cell (Caco-2) monolayer. Three independent experiments were performed within each cell line; n = 3, respectively.

We monitored the transcytotic efficacy of tetracosameric *α*-TTP_*S*_ compared to its monomer form in an *in vitro* transwell model system comprising confluent and maturely developed monolayers of human umbilical vein endothelial cells (HUVECs)^42^ (Fig. 5). Measurements on human transferrin served as positive control as in previous studies^43^. Addition of rhodamine isothiocyanate-labeled dextran simultaneously confirmed the integrity of the HUVEC cell monolayers and served to determine the paracellular flux^42, 43^. Our measurements report a 28-fold and 10-fold increase in the flux through the endothelial cell layer of *α*-TTP_*S*_ and *α*-TTP, respectively, compared to that of rhodamine isothiocyanate-labeled dextran. Our data also show that *α*-TTP_*S*_ crosses the endothelium at a flux rate 9.6 times faster than human transferrin. Repetition of the experiments with polarised epithelial monolayers of Caco-2 cells forming a tight barrier in the same transwell system^44^ did not report any transport for either *α*-TTP_*S*_ or *α*-TTP. This may suggest that the transcytotic transport mechanism for *α*-TTP might be specific to endothelial cells only which warrants further verification.

## Discussion

### Assembly mechanism

Crystallisation trials indicated that *α*-TTP_*S*_ crystals grow with protein concentrations above 12 mg/ml regardless of the chosen condition. On the other hand, we were able to observe the self-assembly of *α*-TTP_*S*_ in solution by optimising the ligand loading process in the presence of anionic detergent at protein concentrations as low as 1 mg/ml of protein. Subjecting soluble preparations to preparative SEC revealed a peak M representing monomeric *α*-TTP and a peak S representing oligomeric *α*-TTP (see Fig. 1A). When analysing fractions of the oligomeric *α*-TTP peak S by native PAGE a sharp band representing *α*-TTP_*S*_ besides oligomers with aggregation number <24 and of monomeric *α*-TTP respectively were detected. Initially, we hypothesised that oligomerisation might be under the control of a reversible equilibrium between the oligomeric states and monomeric *α*-TTP. However, when separating *α*-TTP_*S*_ by analytical SEC from the lower molecular weight oligomers of *α*-TTP no significant re-equilibration of the former state into any other state could be detected by native PAGE (Fig. 1D). We interpreted these results as an evidence that mature *α*-TTP_*S*_ particles represent a kinetically trapped state that does neither re-equilibrate into metastable *α*-TTP oligomers with aggregation number <24 nor into monomeric *α*-TTP. It was concluded that, whenever conditions for early aggregation are satisfied, then the particles can rapidly develop into the final *α*-TTP_*S*_ structure.

We also observed that apo-*α*-TTP in the absence of its cognate ligand is in equilibrium between monomeric, dimeric and tetrameric forms, but it never develops into heavier oligomers (Fig. S1A). The previously determined crystallographic structure of homo-dimeric apo-*α*-TTP shows an intercalated structure where the two mobile gate helices of each monomer protrude into the binding cavity of the other protein^39^. This protein-protein interface is inconsistent with those appearing in *α*-TTP_*S*_, indicating that apo-dimer cannot structurally develop into such oligomer.

Comparison between the X-ray structures of the monomeric ligand complex *α*-TTP^39^ and *α*-TTP_*S*_ shows that the overall fold is well conserved. In particular, the portion of protein surface involved in the tetrameric interface contacts is fully solvent exposed in *α*-TTP. Moreover, no structural changes occur in this area when proteins aggregate in *α*-TTP_*S*_. The ordered part of the trimeric interface in *α*-TTP_*S*_ is not exposed to the solvent due to folding of its N-terminal segment (aa’s 25–47). Comparison of the crystal structure of the monomeric ligand complex of *α*-TTP with known structures of the different members of the Sec-14 like family evidences that the N-terminal segment 1–47 is s not always fully detected by X-ray scattering, it may organise in different conformations^45–48^. This may indicate that this portion of the protein is less structured and more prone to refolding. Formation of trimeric assemblies requires that this region of the protein is displaced in the outer space of *α*-TTP_*S*_ (Fig. 3). In our crystal structure, the 1–47 segment was not detected, probably due to conformational disorder.

The trimeric interface is wider than the tetrameric one (447 Å^2^, and 243 Å^2^, respectively) and characterised by a larger number of strong contact interactions (Fig. 4). Estimation of the binding energy per protein per surface confirms that the trimeric interface is strongly favoured with respect to the tetrameric one (∆*G*_*bind*_(*trimer*) = −4.70 kcal mol^−1^, ∆*G*_*bind*_(*tetramer*) = −3.50 kcal mol^−1^) (Table 2). Structural analysis of the protein contact surfaces by the ProFace server^49, 50^ revealed that the total trimeric interface was 862.46 Å^2^ and includes 4% of the total protein surface area of each protomer, with roughly a third (28%) being fully buried. On the contrary, the tetrameric interface is smaller with 464.32 Å^2^ and not solvent exposed for only its 12%. Surface complementarity^51^ of the two interfaces report 0.7 and 0.62 values, respectively. Summing up these evidences, unmasking of an otherwise inaccessible surface area is a mandatory requirement, besides a cognate bound ligand, for efficient self- assembly of *α*-TTP_*S*_. We propose that natively folded *α*-TTP produces dimers and tetramers by protein-protein interaction through the tetrameric interface. Refolding of the 1–47 N-terminal region activates aggregation through the trimeric interface, which leads to more stable light molecular weight oligomers, which can rapidly evolve into the *α*-TTP_*S*_ assembly.

Native PAGE experiments indicate the presence of multiple oligomeric states with aggregation number <24 (Fig. 1B). Structural comparison between *α*-TTP and *α*-TTP_*S*_ alone cannot elucidate the mechanisms of refolding of the N-terminal segment. In our experiment, we observe that formation of *α*-TTP_*S*_ is facilitated by either the presence of sodium cholate or alternatively by high salt concentrations (i.e. [NH_4_SO_4_] = 100 mM), suggesting that interference of amphiphilic or charged species disturbing the N and C termini triggers the required local refolding. We notice that folding stabilisation of the C-terminal amino acid residues accompanies the maturation of *α*-TTP_*S*_. In the oxidised *α*-TTP_*S*_, it is involved in a strong H-bond and salt-bridge network of interactions with the partner protein (Fig. 3).

### Transcytosis

Receptor-mediated entry mechanisms of macromolecules into barrier-protected tissues have been reported in the last decades for insulin^52^, immunoglobulin^53^, low density lipoprotein^54^ and ferritin^55^. Here we report for the first time the existence of a tetracosameric functional transcytotic unit of *α*-TTP. Very recently, the existence of oligomeric states functional for lipid transfer have been postulated for Sec-14 like proteins by the groups of Arai and Bankaitis^16, 56^.

*In vitro* measurements of transcytotic flux evidence that *α*-TTP_*S*_ efficiently and selectively transfers mature HUVEC monolayers. Compared to transferrin, serving as positive control^43, 57, 58^, the transfer rate of *α*-TTP_*S*_ in HUVEC cells is significantly (9.6-fold) faster. The choice of transferrin as positive control system is justified by evidences of its receptor-mediated transcytosis in a broad range of cell culture models (L-6 cells, HMVEC-d, HeLa, 293 T)^57–60^. In particular, transferrin flux rates in HUVEC cell monolayers found by us are similar to those measured through a bovine retinal endothelial cell (BREC) monolayer^42^.

Interestingly, ferritin, the iron storage protein comprising 24 monomers exerted similar flux rates in the BREC model as transferrin^43^. *α*-TTP_*S*_ has similar topology as the 24-meric assembly of ferritin^61^, which is involved in homoeostasis of iron in the brain. The two proteins are not homologous. Thus our finding indicates convergent evolution towards a specific protein assembling topology that facilitates controlled endothelial crossing. Contrary to endothelia, *α*-TTP_*S*_ transfer rates in human Caco-2 epithelial cells are barely detectable, indicating the existence of an efficient and directed transport system for *α*-TTP through endothelial cell layers. Nevertheless, a lack of transcytosis does not necessarily mean that Caco-2 cells do not take up TTP or export vitamin E, but putatively less effectively or *via* other mechanisms. Grebenstein and co-workers demonstrated that in TTP knockout mice fed equimolar doses of *α*- and *γ*-tocopherol *α*-TTP knockout resulted in almost complete depletion of *α*-Tol in the brain, but not in many other tissues. These data suggest a rather tissue-specific delivery of *α*-Tol *via* TTP thereby maintaining *α*-Tol concentrations within a certain range^62^. On the basis of recent animal studies it has been proposed that *α*-TTP is essential for the bioavailability and the delivery of *α*-Tol to specific target sites in humans^23, 63^. Deletion of the *α*-TTP gene in mice resulted in a reduced accumulation of dietary *α*-Tol in the liver and depletion of peripheral tissue *α*-Tol while the brain was the organ that was affected most significantly^64^. Traber and co-workers describe an *α*-TTP-mediated preferential enrichment of *α*-Tol in very low density lipoproteins in dependency of metabolic processes in the liver (20, 35) and the *α*-Tol-associated antioxidative protection of neurones (23, 27). Anyway, tissue-specificity and the fascinating hypothesis of a conceivable receptor-mediated *α*-TTP_*S*_ transport has to be explored by future studies using further endothelial and epithelia cell models mimicking different compartments of the organism.

### Perspectives

By its own structure, *α*-TTP acquires several properties including substrate recognition and binding, self-assembly, auto cross-linking, and transcytotic potency. Intrinsic to the assembly, inhibition of substrate release by steric blockage of the mechanism of access to the ligand binding cavity also occurs. The discovery of such an intrinsic ability to self-assemble into functional nano-particles paves the way to future studies determining the means by which vitamin E is physiologically trafficked through the body.

Moreover, regardless of its potential physiological role, discovery of an endogenous nano-structure featuring such properties opens large perspectives towards new bio-medical/biotechnological applications. The cavity present in *α*-TTP_*S*_ could in principle accommodate nucleic acids, proteins or lipid fractions of suitable size and charge distribution. For this, potential substrates would have to be encapsulated during oligomer assembly or, alternatively, travel through the pores to reach the inner cavity. The eventual presence of lipids in the same cavity may also facilitate localisation of generic lipophilic substrates. The discovery of an endogenous transport protein of *α*-Tol having intrinsic capability of crossing endothelial cells may open to new ways of drug delivery. Finally, eventual identification and characterisation of a receptor for *α*-TTP_*S*_ may lead to engineering selective entry pathways to specific tissues protected by endothelia such as brain, placenta or others.

## Methods

### Expression and Purification

The N-terminal (His)_6_-tagged *α*-TTP expression construct was made by cloning the PCR product derived from a human cDNA library into the NdeI and XhoI sites of the pET-28a vector (Stratagene, CA. USA) using the primers 5′- GGGAATTCGCAGAGGCGCGATCCCAG -3′ and 5′- CCGTCATTGAATGCTCTCAGAAATGC -3′. Protein expression was performed in *Escherichia coli* strain BL21(DE3) under control of the T7 promoter. Transformed bacteria were grown at 37 °C to an OD_600_ of 0.8 and induced with 330 *µ*M isopropyl-thiogalactopyranoside (IPTG) overnight at 30 °C. Bacteria were harvested by centrifuging at 7300 g and 4 °C for 30 minutes. Bacterial pellets obtained from one liter of medium were re-suspended in 25 ml lysis buffer (20 mM Tris, pH 8.0, 100 mM NaCl, 10 mM imidazole, 0.5 (v/v) Triton X-100 and 1 mM phenylmethylsulfonyl fluoride). Harvested cells were disrupted twice in a French pressure cell. The lysate was centrifuged at 39000 g and 4 °C for 40 minutes. The clarified supernatant was passed through a column containing 12 ml of TALON Superflow (Clontech Laboratories, CA, USA). Non-specifically bound proteins were removed by rinsing the column with washing buffer (20 mM Tris, 100 mM NaCl, 10 mM imidazole, pH 8.0) until the UV absorption at 280 nm recovered the level of the base line. The protein was eluted with elution buffer (20 mM Tris, 100 mM NaCl, 150 mM imidazole, pH 8.0). The (His)_6_-tag was cleaved off using thrombin (GE Healthcare, Little Chalfont, UK) in elution buffer (20 mM Tris, 100 mM NaCl, 150 mM imidazole, pH 8.0) at 4 °C overnight. The protein eluate was pooled and concentrated using Vivaspin (Sartorius, Gottingen, DE) centrifugal concentrators (MWCO 10 kDa) to ≤2.5 mg/ml in order to prevent aggregation of apo-*α*-TTP.

### Preparation of *α*-TTP ligand-complexes

Protein-ligand complex formation was induced by dialysing freshly prepared apo-*α*-TTP in the presence of detergent solubilised *α*-Tol. In brief, a droplet of 1 mg of *α*-Tol was overlaid with 40.9 mg of solid sodium cholate and subsequently suspended in 1 ml of elution buffer (20 mM Tris, 100 mM NaCl, 150 mM imidazole, pH 8.0). The suspension was bath sonicated until all material had dissolved to a clear solution. Apo-*α*-TTP (11 ml at ≤2.5 mg/ml) was complemented with the tocopherol-sodium cholate solution at 9:1 (v/v) ratio and transferred into a CelluSep T3 dialysis tubular membrane with an MWCO range of 12–14 kDa (Membranes Filtration Products. TX, USA). Dialysis was performed in two steps against 3 l buffer (20 mM Tris, 100 mM NaCl, pH 8.0) each for six hours at 4 °C. The dialysate (12 ml) was filtered through a Millex GP 0.22 *µ*m filter (EMD Milipore, MA, USA), supplemented with Triton X-100 at a final concentration of 0.01% (v/v), reduced to 2 ml and separated by preparative size exclusion chromatography (SEC) (Fig. S1). Fractions corresponding to the size of the monomeric *α*-TTP ligand-complex were pooled and concentrated to 20 mg/ml using Vivaspin concentrators (MWCO 10 kDa; Sartorius, Gottingen, DE) and directly used for crystallisation. Fractions corresponding to the size of *α*-TTP_*S*_ nano-spheres were pooled and concentrated using Vivaspin concentrators (MWCO 30 kDa) to 10 mg/ml re-purified by analytical SEC.

### Crystallisation and structure determination of *α*-TTP_*S*_

Crystals were grown by either hanging or sitting-drop vapour diffusion using reservoir solutions ranging from 10 to 15% PEG-4000, 100–175 mM ammonium sulphate in 100 mM Hepes sodium pH 7.5 at 18 °C. Freshly prepared monomeric *α*-TTP ligand-complex was used in a concentration range between 12–22 mg/ml. Highest quality crystals of fully reduced *α*-TTP_*S*_ were observed within two weeks at drop ratios of protein over reservoir ranging between 3/1 and 2/1 (v/v). Crystals had cubic shape with edge length ranging between 20 and 80 *µ*M. Isomorphous crystals of fully oxidised *α*-TTP_*S*_ were collected after two months. All crystals were flash frozen in nitrogen after adding glycerol in two steps to a final concentration of 20% (v/v). Diffraction data were collected at the Swiss Light Source (SLS) synchrotron beamline X06DA (PSI Villigen) at 100 K, employing a Dectris Pilatus 2 M CCD detector (DECTRIS Ltd., Baden, Switzerland). All data were indexed, integrated and scaled with XDS^65^. Phaser-MR was used for calculating the initial phases with the truncated structure model (residues 47–275) of monomeric *α*-TTP (PDB ID: 1OIP) as search structure. The atomic models of reduced *α*-TTP_*S*_ and of oxidised *α*-TTP_*S*_ were both refined by iterative cycles of manual model building using COOT^66^ and restrained refinements using the Phenix program suite^67^. Coordinates and structure factors of both structures have been deposited in the RCSB Protein Data Bank with ID codes 5MUE and 5MUG.

### Binding energy calculations

The oligomerisation energies were computed for the trimeric and tetrameric interfaces using standard thermodynamic solvation/association cycle as described in ref. 68. The solvation energy was computed by numerical solution of the Linearized Poisson-Boltzmann equation using APBS^69^. Both the charge distribution and the non-electrostatic part of the binding energy were estimated using the AMBER force field parameters described by Maier *et al*.^70^.

### Size exclusion chromatography

Preparative (Fig. S2B) and analytical SEC (Fig. S1) of *α*-TTP_*S*_ oligomers was performed on HiLoad 16/60 Superose 75 prep grade and on Superose 6 10/300 columns respectively (GE Healthcare, Little Chalfont, UK), both attached to an ÄKTA Purifier chromatography system (GE Healthcare, Little Chalfont, UK). Runs were performed in SEC buffer (10 mM Tris, 100 mM NaCl, pH 8.0) at flow rates ranging from 0.5 (analytical) to 1.5 ml/minute (preparative) at 6 °C. Both SEC columns were calibrated using commercially available protein calibration kits (GE Healthcare, Little Chalfont, UK). MALS experiments were performed in 10 mM Tris pH 8.0, 100 mM NaCl, using a Superose 6 Increase 10/300 GL analytical size exclusion chromatography column (GE Healthcare, Little Chalfont, UK) connected in line to mini-DAWN TREOS light scattering and Optilab T-rEX refractive index detectors (Wyatt Technology, CA, USA). Monomeric *α*-TTP was injected at a concentration of 485 *µ*M; the oligomeric *α*-TTP_*S*_ at a concentration of 36.6 *µ*M.

### Negative-stain transmission electron microscopy

A sample of *α*-TTP_*S*_ at a concentration of 300 *µ*g/ml was adsorbed for 1 minute to parlodion carbon-coated copper grids, which were previously rendered hydrophilic by glow discharge at low pressure in air. After adsorption the grids were washed with three drops of double-distilled water and stained with two drops of 0.75% uranyl formate. Electron micrographs were recorded with a Philips CM12 transmission electron microscope operated at 80 kV and equipped with a Morada CCD camera (Soft Imaging System). Image analysis was performed with the ImageJ image processing program V1.49o (NIH, MD, USA).

### Native polyacrylamide gel electrophoresis

Native PAGE was performed using pre-cast NativePAGE Novex 4–16% Bis-Tris Protein Gels (Life Technologies, CA, USA). Each protein sample (20 *µ*l, 0.5 mg/ml) was mixed with an equal volume of native-PAGE loading buffer (62 mM Tris, 25% glycerol, 1% bromophenol blue, pH 6.8). Gels were run at 4 °C in running buffer (50 mM Tricine, 50 mM BisTris, pH 8.0) at 160 V for 30 minutes and then at 180 V until the bromophenol blue marker reached the end of the gel. Protein visualisation was achieved by staining with SYPRO ruby protein gel stain (Life Technologies, CA, USA).

### Western Blotting

In brief, SDS-PAGE was carried out on 12% PAGE gels. Before blotting on nitrocellulose membranes gels were incubated for 20 minutes in transfer buffer (25 mM Tris, 192 mM glycine, 20% methanol, pH 8.3). Blotting was carried out at 130 mA for 50 minutes using a semi-dry blotting apparatus (Bio-Rad Laboratories, CA, USA). For analysis a commercially available primary antibody against *α*-TTP (alpha TTP Antibody [C2C3] C-term, GeneTex Inc., CA, USA) was used. IRDye secondary antibodies from LI-COR were employed for visualisation with either IRDye 800CW or IRDye 680RD and scans were performed on a LI-COR Odyssey infrared system (LI-COR Biosystems, NE, USA) (Fig. S2D).

### Dynamic light scattering

Freshly pooled fractions of *α*-TTP (concentration range 0.1–0.2 mg/ml) obtained from analytical SEC (10 mM Tris, 100 mM NaCl, pH 8.0) corresponding to *α*-TTP_*S*_ were analysed by dynamic light scattering (DLS). Determination of the size distribution profile of each sample was performed on a DynaPro molecular sizing instrument (Protein-Solutions) using UVettes^®^ (Eppendorf, Hamburg, DE) of 1 cm path length. Each data set was collected for at least 5 minutes containing a minimum of 100 single measurements.

### Thermodynamic analysis of *α*-TTP and *α*-TTP_*S*_

A Jasco J-175 Spectropolarimeter with a Peltier PFD-350S temperature controller was used to monitor temperature-depended protein unfolding of apo-*α*-TTP, monomeric *α*-TTP and of *α*-TTP_*S*_. For this, a 1 mm path length quartz cell (with 100 *µ*l sample) was used, and the protein concentration ranged from 0.1–0.5 mg/ml. The response was set to 1 s with a bandwidth of 5 nm. Following the results from the CD spectra, the wavelength was adjusted to 222 nm for temperature-dependent protein unfolding experiments. The temperature was increased at a rate of 2 K min^−1^ from 20 °C to 80 °C, for monomeric *α*-TTP, and from 20 °C to 100 °C, for *α*-TTP_*S*_, both in increments of 0.5 K. The transition temperatures (T_*m*_) were calculated from the 1st derivative of the unfolding curves.

### Transcytosis

*α*-TTP fractions corresponding to monomeric and tetracosameric protein from analytic gel filtration were labelled with fluorescein isothiocyanate (FITC) according to a method previously described by Harlow *et al*.^71^. In brief, protein samples were transferred into carbonate buffer (0.1 M, pH 9.0) for labelling using PD-10 desalting columns (GE Healthcare, Little Chalfont, UK) previously equilibrated in the same buffer. FITC was freshly dissolved before use in anhydrous DMSO (1 mg/ml). The labelling reaction was started by adding 50 *µ*l of FITC DMSO solution to one ml of protein (1 mg/ml). The reaction mixture was incubated for 2 hours at 37 °C and stopped by removing excessive FITC using a PD-10 column previously equilibrated in PBS (10 mM phosphate, 138 mM NaCl, 27 mM KCl, pH 7.4). The labelled protein samples were finally purified by analytical GFC on a Superose 6 10/300 GL column (GE Healthcare, Little Chalfont, UK) in PBS. Transferrin was labelled by the same method and used as positive control in transcytosis experiments. In order to demonstrate promotion of transcytosis by *α*-TTP_*S*_ oligomers we used primary endothelial cells from human umbilical veins (HUVECs). HUVECs were isolated from fresh umbilical cords with the help of trypsin/EDTA according to a Miltenyi Biotec protocol^72^. The authenticity of endothelial origin was verified via positive immunofluorescence co-staining of von Willebrand factor (vWF) and CD31. HUVECs were cultured in a transwell system (permeable polyester membranes with 0.4 *µ*m pore size; Corning, USA) in Endothelial Cell Growth Medium (Promocell, Germany) comprising 100 U/ml penicillin and 100 *µ*g/ml streptomycin (PAN Biotech, Germany) with gelatin pre-coating (Sigma-Aldrich, Germany). Cells were allowed to form a tight monolayer within 7 days of culture while medium was changed every other day. For transcytosis measurements medium comprising 200 *µ*g of RITC-dextran 70 kDa (Sigma Aldrich, MO, USA) and either 200 *µ*g of FITC labelled monomeric *α*-TTP, oligomeric *α*-TTP_*S*_ or transferrin (as a positive control) was applied to the apical chamber, respectively. Transport was monitored by sampling 100 *µ*l of basolateral medium at various time points (15, 30, 45, 60, 120, 180, and 240 min) after addition of samples to the apical chamber. Basolateral aliquots were subsequently analysed for fluorescence with a Tecan infinite200 microplate reader (Tecan, Mannedorf, CH) at an excitation wavelength of 485 nm and an emission wavelength of 535 nm (FITC) followed by measurements at an excitation wavelength of 535 nm and an emission wavelength of 590 nm (RITC), respectively. The Caco-2/TC7 cell line (human colorectal adenocarcinoma cells; kindly provided by Dr G. Lietz, Newcastle University, UK) representing an epithelial cell model was used as a negative control in transcytosis experiments. Caco-2 cells were maintained in Dulbecco,s Modified Eagles Medium containing 4.5 g/l glucose, 4 mmol/l L-glutamine, 1 mmol/l sodium pyruvate, 100/ml penicillin, 100 *µ*g/ml streptomycin (PAN Biotec, Germany) and 20% (v/v) FCS (Gibco, Germany). Caco-2 cells are widely used as an *in vitro* model for barrier and transport studies^44, 73, 74^. Caco-2 cells differentiate and form a tight epithelial monolayer in the same transwell system as described above within 14 days of culture. The differentiation process of Caco-2 cells is well-characterised and can be measured by various markers: increase in TEER and alkaline phosphatase activity, cellular dome formation^75^, tight junction formation, polarisation, development of apical microvilli^76^ and reduced permeability already within 4 days of differentiation. The Caco-2/TC-7 clone used in this study differentiates faster than the parental Caco-2 cell line, observed by a more rapid increase in TEER values^77^. TEER directly correlates with differentiation state as well as barrier tightness of Caco-2 cells^78^. The higher transepithelial resistance, calculated in Ω/cm^2^, the higher tightness and the lower paracellular permeability of Caco-2 cell monolayers, thus, transport remains exclusive for the transcellular route^79^. Therefore, TEER measurement is a suitable, non-invasive method to longitudinally evaluate barrier tightness of Caco-2 cells in response to compounds provided via the apical side of the monolayer resembling the luminal side of the gut. In our experiments, differentiation was confirmed by TEER measurements. Caco-2/TC-7 monolayers were cultured for 14 days while TEER increased to 388.8 6.1 Ω/cm^2^ due to the differentiation process. As initial TEER values of confluent, but non-differentiated Caco-2/TC-7 monolayers, are averagely classified by 96.4 ± 6.8 Ω/cm^2^, differentiation process was well advanced, indicated by a 403.4 ± 6.3% increase of initial TEER values. Although Caco-2 cells seem to be able to absorb tocopherols indicated by influencing stress-activated signal transduction pathways^80^ bioavailability is markedly low^81^. Thereby tocopherols pass intestinal epithelium solely following transfer into mixed micelles and incorporation into chylomicrons for trans-epithelial transport to the lymphatic and blood system^82, 83^.

Transcytosis experiments were performed according to the HUVEC experiments. The rate of flux was calculated as previously described by Fisher *et al*.^43^ using equation (1).

1 $$\frac{\frac{{B}_{f}}{{A}_{f}}\times \frac{{V}_{b}}{A}}{t}=J$$

The flux is here the slope (cm s^−1^) of the basolateral fluorescence (*B*_*f*_) per unit amount of apical fluorescence (*A*_*f*_) normalised to the volume of basolateral chamber (*V*_*b*_) and to the area available for transport (*A*) against time (*t*). As a control for paracellular flux and as assurance for the formation of tight junctions, rhodamine isothiocyanate (RITC) dextran (70 kDa) was added simultaneously to the apical chamber in each experiment as tight junction control. The level of paracellular transport by RITC dextran was measured in the same manner as FITC-*α*-TTP, except that the RITC was detected at an excitation wavelength of 545 nm and an emission wavelength of 590 nm respectively. The different fluorescence behaviour of RITC and FITC has allowed for the simultaneous analysis of the protein of interest and the dextran control. Since dextran is not internalised at appreciable levels by endothelial cells, any accumulation of dextran in the basal chamber correlates with paracellular flux. Three independent transcytosis experiments were performed using HUVEC and Caco-2 cell monolayers, respectively.

## Electronic supplementary material

Supplementary Information

## References

[1] Evans, H. M. & Bishop, K. S. On the existence of a hitherto unrecognised dietary factor essential for reproduction. *Science* 650–651 (1922). 10.1126/science.56.1458.650 17838496

[2] Mene-Saffran‘ e L, Jones AD, DellaPenna D´ (2010). Plastochromanol-8 and tocopherols are essential lipid-soluble antioxidants during seed desiccation and quiescence in arabidopsis. Proceedings of the National Academy of Sciences.

[3] Kayden HJ, Traber MG (1993). Absorption, lipoprotein transport, and regulation of plasma concentrations of vitamin E in humans. Journal of lipid research.

[4] Burton GW (1998). Human plasma and tissue alpha-tocopherol concentrations in response to supplementation with deuterated natural and synthetic vitamin e. The American journal of clinical nutrition.

[5] Jiang Q, Elson-Schwab I, Courtemanche C, Ames BN (2000). *γ*-tocopherol and its major metabolite, in contrast to *α*-tocopherol, inhibit cyclooxygenase activity in macrophages and epithelial cells. Proceedings of the National Academy of Sciences.

[6] Wang X (2006). Mechanism of arylating quinone toxicity involving michael adduct formation and induction of endoplasmic reticulum stress. Proceedings of the National Academy of Sciences of the United States of America.

[7] Burton GW, Joyce A, Ingold KU (1983). Is vitamin e the only lipid-soluble, chain-breaking antioxidant in human blood plasma and erythrocyte membranes?. Archives of Biochemistry and Biophysics.

[8] Ingold K (1987). Vitamin e remains the major lipid-soluble, chain-breaking antioxidant in human plasma even in individuals suffering severe vitamin e deficiency. Archives of biochemistry and biophysics.

[9] Liebler D, Kling D, Reed D (1986). Antioxidant protection of phospholipid bilayers by alpha-tocopherol. control of alpha-tocopherol status and lipid peroxidation by ascorbic acid and glutathione. Journal of Biological Chemistry.

[10] Krieger-Liszkay A, Trebst A (2006). Tocopherol is the scavenger of singlet oxygen produced by the triplet states of chlorophyll in the psii reaction centre. Journal of experimental botany.

[11] Bankaitis VA, Aitken JR, Cleves AE, Dowhan W (1990). An essential role for a phospholipid transfer protein in yeast golgi function. Nature.

[12] Bankaitis VA, Malehorn DE, Emr SD, Greene R (1989). The saccharomyces cerevisiae sec14 gene encodes a cytosolic factor that is required for transport of secretory proteins from the yeast golgi complex. The Journal of Cell Biology.

[13] Arita M (1995). Human alpha-tocopherol transfer protein: cdna cloning, expression and chromosomal localization. Biochemical Journal.

[14] Min KC, Kovall RA, Hendrickson WA (2003). Crystal structure of human *α*-tocopherol transfer protein bound to its ligand: implications for ataxia with vitamin e deficiency. Proceedings of the National Academy of Sciences.

[15] Helbling RE, Aeschimann W, Simona F, Stocker A, Cascella M (2012). Engineering tocopherol selectivity in *α*-ttp: A combined *In Vitro/In Silico* study. PLoS ONE.

[16] Kono N (2013). Impaired α-ttp-pips interaction underlies familial vitamin e deficiency. Science.

[17] Oram JF, Vaughan AM, Stocker R (2001). Atp-binding cassette transporter a1 mediates cellular secretion of *α*-tocopherol. Journal of Biological Chemistry.

[18] Horiguchi M (2003). ph-dependent translocation of *α*-tocopherol transfer protein (*α*-ttp) between hepatic cytosol and late endosomes. Genes to Cells.

[19] Qian J (2005). Intracellular trafficking of vitamin e in hepatocytes: the role of tocopherol transfer protein. Journal of lipid research.

[20] Traber MG, Burton G, Hamilton RL (2004). Vitamin e trafficking. Annals of the New York Academy of Sciences.

[21] Traber M (1990). Impaired ability of patients with familial isolated vitamin e deficiency to incorporate alpha-tocopherol into lipoproteins secreted by the liver. Journal of Clinical Investigation.

[22] Jishage K-i (2001). *α*-tocopherol transfer protein is important for the normal development of placental labyrinthine trophoblasts in mice. Journal of Biological Chemistry.

[23] Miller GW (2012). The *α*-tocopherol transfer protein is essential for vertebrate embryogenesis. PloS one.

[24] Traber MG (1987). Lack of tocopherol in peripheral nerves of vitamin e-deficient patients with peripheral neuropathy. New England Journal of Medicine.

[25] Ouahchi K (1995). Ataxia with isolated vitamin e deficiency is caused by mutations in the *α*–tocopherol transfer protein. Nature genetics.

[26] Terasawa Y (2000). Increased atherosclerosis in hyperlipidemic mice deficient in *α*-tocopherol transfer protein and vitamin e. Proceedings of the National Academy of Sciences.

[27] Yokota T (2001). Delayed-onset ataxia in mice lacking *α*-tocopherol transfer protein: model for neuronal degeneration caused by chronic oxidative stress. Proceedings of the National Academy of Sciences.

[28] Traber MG, Atkinson J (2007). Vitamin e, antioxidant and nothing more. Free Radical Biology and Medicine.

[29] Spector R, Johanson CE (2007). Review: Vitamin transport and homeostasis in mammalian brain: focus on vitamins b and e. Journal of neurochemistry.

[30] Kaempf-Rotzoll D (2002). *α*-tocopherol transfer protein is specifically localized at the implantation site of pregnant mouse uterus. Biology of reproduction.

[31] Jauniaux E (2004). Distribution and transfer pathways of antioxidant molecules inside the first trimester human gestational sac. The Journal of Clinical Endocrinology & Metabolism.

[32] Lamprakis, C., Stocker, A. & Cascella, M. Mechanisms of recognition and binding of *α*-ttp to the plasma membrane by multi-scale molecular dynamics simulations. *Frontiers in Molecular Biosciences* **2**, 36 (2015). 10.3389/fmolb.2015.00036 PMC4487086 26191529

[33] Chung S (2016). Vitamin e and phosphoinositides regulate the intracellular localization of the hepatic *α*-tocopherol transfer protein. Journal of Biological Chemistry.

[34] Arita M, Nomura K, Arai H, Inoue K (1997). *α*-tocopherol transfer protein stimulates the secretion of *α*-tocopherol from a cultured liver cell line through a brefeldin a-insensitive pathway. Proceedings of the National Academy of Sciences.

[35] Shichiri M (2010). Atp-binding cassette transporter a1 is involved in hepatic *α*-tocopherol secretion. The Journal of nutritional Biochemistry.

[36] Traber MG, Burton GW, Ingold KU, Kayden HJ (1990). Rrr- and srr-alpha-tocopherols are secreted without discrimination in human chylomicrons, but rrr-alpha-tocopherol is preferentially secreted in very low density lipoproteins. Journal of Lipid Research.

[37] Traber MG, Arai H (1999). Molecular mechanisms of vitamin e transport. Annual review of nutrition.

[38] Tam JP, Wu CR, Liu W, Zhang JW (1991). Disulfide bond formation in peptides by dimethyl sulfoxide. scope and applications. Journal of the American Chemical Society.

[39] Meier R, Tomizaki T, Schulze-Briese C, Baumann U, Stocker A (2003). The molecular basis of vitamin e retention: structure of human *α*-tocopherol transfer protein. Journal of molecular biology.

[40] Clackson T, Wells JA (1995). A hot spot of binding energy in a hormone-receptor interface. Science.

[41] Wells JA (1996). Binding in the growth hormone receptor complex. Proceedings of the National Academy of Sciences.

[42] Nevo N (2001). Increasing endothelial cell permeability improves the efficiency of myocyte adenoviral vector infection. The journal of gene medicine.

[43] Fisher J (2007). Ferritin: a novel mechanism for delivery of iron to the brain and other organs. American Journal of Physiology-Cell Physiology.

[44] Piegholdt S (2014). Biochanin a and prunetin improve epithelial barrier function in intestinal Caco-2 cells via downregulation of erk, nf-*κ*b, and tyrosine phosphorylation. Free Radical Biology and Medicine.

[45] Sha B, Phillips SE, Bankaitis VA, Luo M (1998). Crystal structure of the saccharomyces cerevisiae phosphatidylinositoltransfer protein. Nature.

[46] He X, Lobsiger J, Stocker A (2009). Bothnia dystrophy is caused by domino-like rearrangements in cellular retinaldehydebinding protein mutant r234w. Proceedings of the National Academy of Sciences.

[47] Christen M (2015). Structural insights on cholesterol endosynthesis: Binding of squalene and 2, 3-oxidosqualene to supernatant protein factor. Journal of structural biology.

[48] Ren J (2014). A phosphatidylinositol transfer protein integrates phosphoinositide signaling with lipid droplet metabolism to regulate a developmental program of nutrient stress–induced membrane biogenesis. Molecular biology of the cell.

[49] Saha RP, Bahadur RP, Pal A, Mandal S, Chakrabarti P (2006). Proface: a server for the analysis of the physicochemical features of protein-protein interfaces. BMC structural biology.

[50] Bahadur RP, Chakrabarti P, Rodier F, Janin J (2004). A dissection of specific and non-specific protein–protein interfaces. Journal of molecular biology.

[51] Lawrence MC, Colman PM (1993). Shape complementarity at protein/protein interfaces. Journal of molecular biology.

[52] Duffy KR, Pardridge WM (1987). Blood-brain barrier transcytosis of insulin in developing rabbits. Brain research.

[53] Perez J, Branch W, Smith L, Mullock B, Luzio J (1988). Investigation of endosomal compartments involved in endocytosis and transcytosis of polymeric immunoglobulin a by subcellular fractionation of perfused isolated rat liver. Biochemical Journal.

[54] Dehouck B (1997). A new function for the ldl receptor: transcytosis of ldl across the blood–brain barrier. The Journal of cell biology.

[55] Tuma PL, Hubbard AL (2003). Transcytosis: crossing cellular barriers. Physiological Reviews.

[56] Ghosh R (2015). Sec14-nodulin proteins and the patterning of phosphoinositide landmarks for developmental control of membrane morphogenesis. Molecular biology of the cell.

[57] Harila K, Salminen A, Prior I, Hinkula J, Suomalainen M (2007). The vpu-regulated endocytosis of hiv-1 gag is clathrinindependent. Virology.

[58] Raghu H, Sharma-Walia N, Veettil MV, Sadagopan S, Chandran B (2009). Kaposi’s sarcoma-associated herpesvirus utilizes an actin polymerization-dependent macropinocytic pathway to enter human dermal microvascular endothelial and human umbilical vein endothelial cells. Journal of Virology.

[59] Fishman J, Rubin J, Handrahan J, Connor J, Fine R (1987). Receptor-mediated transcytosis of transferrin across the blood-brain barrier. Journal of neuroscience research.

[60] Roberts RL, Fine RE, Sandra A (1993). Receptor-mediated endocytosis of transferrin at the blood-brain barrier. Journal of Cell Science.

[61] Lawson, D. M. *et al*. Solving the structure of human h ferritin by genetically engineering intermolecular crystal contacts. *Nature* **349**, 541-4 (1991). 10.1038/349541a0 1992356

[62] Grebenstein N, Schumacher M, Graeve L, Frank J (2014). α‐Tocopherol transfer protein is not required for the discrimination against γ‐tocopherol *in vivo* but protects it from side‐chain degradation *in vitro*. Molecular nutrition & food research.

[63] Choi J (2015). Novel function of vitamin e in regulation of zebrafish (danio rerio) brain lysophospholipids discovered using lipidomics. Journal of lipid research.

[64] Leonard SW, Terasawa Y, Farese RV, Traber MG (2002). Incorporation of deuterated RRR-or all-rac-α-tocopherol in plasma and tissues of α-tocopherol transfer protein–null mice. The American journal of clinical nutrition.

[65] Kabsch WX (2010). Acta Crystallographica Section D: Biological Crystallography.

[66] Emsley P, Lohkamp B, Scott WG, Cowtan K (2010). Features and development of coot. Acta Crystallographica Section D: Biological Crystallography.

[67] Adams PD (2010). Phenix: a comprehensive python-based system for macromolecular structure solution. Acta Crystallographica Section D: Biological Crystallography.

[68] Fogolari F, Brigo A, Molinari H (2002). The poisson–boltzmann equation for biomolecular electrostatics: a tool for structural biology. Journal of Molecular Recognition.

[69] Baker NA, Sept D, Joseph S, Holst MJ, McCammon JA (2001). Electrostatics of nanosystems: application to microtubules and the ribosome. Proceedings of the National Academy of Sciences.

[70] Maier JA (2015). ff14sb: improving the accuracy of protein side chain and backbone parameters from ff99sb. Journal of chemical theory and computation.

[71] Harlow, E. & Lane, D. Antibodies: A Laboratory Manual (Cold Spring Harbor Laboratory Press New York, 1988).

[72] CD31 MicroBead Kit for the isolation of human microvascular and umbilical vein endothelial cells http://www.miltenyibiotec.com/~/media/Images/Products/Import/0001300/IM0001346.ashx (Date of access: 10/12/2013) (2010).

[73] Kops SK, West AB, Leach J, Miller RH (1997). Partially purified soy hydrolysates retard proliferation and inhibit bacterial translocation in cultured c2bbe cells. The Journal of nutrition.

[74] Levy E, Mehran M, Seidman E (1995). Caco-2 cells as a model for intestinal lipoprotein synthesis and secretion. The FASEB Journal.

[75] Hara A (1993). Changes of proliferative activity and phenotypes in spontaneous differentiation of a colon cancer cell line. Japanese journal of cancer research.

[76] Herold G, Rogler G, Rogler D, Stange EF (1994). Morphology of Caco-2 cells varies in different cell batches. In Vitro Cellular & Developmental Biology-Animal.

[77] Gres M-C (1998). Correlation between oral drug absorption in humans, and apparent drug permeability in tc-7 cells, a human epithelial intestinal cell line: comparison with the parental Caco-2 cell line. Pharmaceutical research.

[78] Grasset E, Pinto M, Dussaulx E, Zweibaum A, Desjeux J (1984). Epithelial properties of human colonic carcinoma cell line Caco-2: electrical parameters. American Journal of Physiology-Cell Physiology.

[79] Farrell TL, Poquet L, Dew TP, Barber S, Williamson G (2012). Predicting phenolic acid absorption in Caco-2 cells: a theoretical permeability model and mechanistic study. Drug Metabolism and Disposition.

[80] Elisia I, Kitts DD (2015). Tocopherol isoforms (*α*-, *γ*-, and *δ*-) show distinct capacities to control nrf-2 and nf*κ*b signaling pathways that modulate inflammatory response in Caco-2 intestinal cells. Molecular and cellular biochemistry.

[81] Desmarchelier C (2013). The distribution and relative hydrolysis of tocopheryl acetate in the different matrices coexisting in the lumen of the small intestine during digestion could explain its low bioavailability. Molecular nutrition & food research.

[82] Failla ML, Chitchumronchokchai C, Ferruzzi MG, Goltz SR, Campbell WW (2014). Unsaturated fatty acids promote bioaccessibility and basolateral secretion of carotenoids and *α*-tocopherol by Caco-2 cells. Food & function.

[83] Nicod N, Parker RS (2013). Vitamin e secretion by Caco-2 monolayers to apoa1, but not to hdl, is vitamer selective. The Journal of nutrition.

[84] Krissinel E, Henrick K (2007). Inference of macromolecular assemblies from crystalline state. Journal of Molecular Biology.

