# Supplementary material for: Self-assembled α-Tocopherol Transfer Protein Nanoparticles Promote Vitamin E Delivery Across an Endothelial Barrier
Source: Sci Rep. 2017 Jul 10;7:4970. doi: 10.1038/s41598-017-05148-9 (PMC5504013; doi:10.1038/s41598-017-05148-9)
Supplement: Supplementary file 1 — Supplementary Information [file 41598_2017_5148_MOESM1_ESM.pdf]

Supplementary Information

Self-assembled α-Tocopherol Transfer Protein Nanoparticles Promote Vitamin E Delivery Across an Endothelial Barrier

Walter Aeschimann[1], Stefanie Staats[2], Stephan Kammer[1], Natacha Olieric[3], Jean-Marc Jeckelmann[4], Dimitrios Fotiadis[4], Thomas Netscher[5], Gerald Rimbach[2], Michele Cascella[6,*] and Achim Stocker[1,+]

[1]University of Bern, Department of Chemistry and Biochemistry, Bern, Switzerland
[2]University of Kiel, Institute of Human Nutrition and Food Science, Kiel, Germany
[3]Paul Scherrer Institut, Villigen, Switzerland
[4]University of Bern, Institute of Biochemistry and Molecular Medicine, Bern, Switzerland
[5]DSM Nutritional Products Ltd., Basel, Switzerland
[6]University of Oslo, Department of Chemistry and Centre for Theoretical and Computational Chemistry (CTCC), Oslo, Norway

[*]michele.cascella@kjemi.uio.no
[+]achim.stocker@dcb.unibe.ch

SUPPLEMENTARY INFORMATION includes:

Supplementary Figures S1, S2, S3

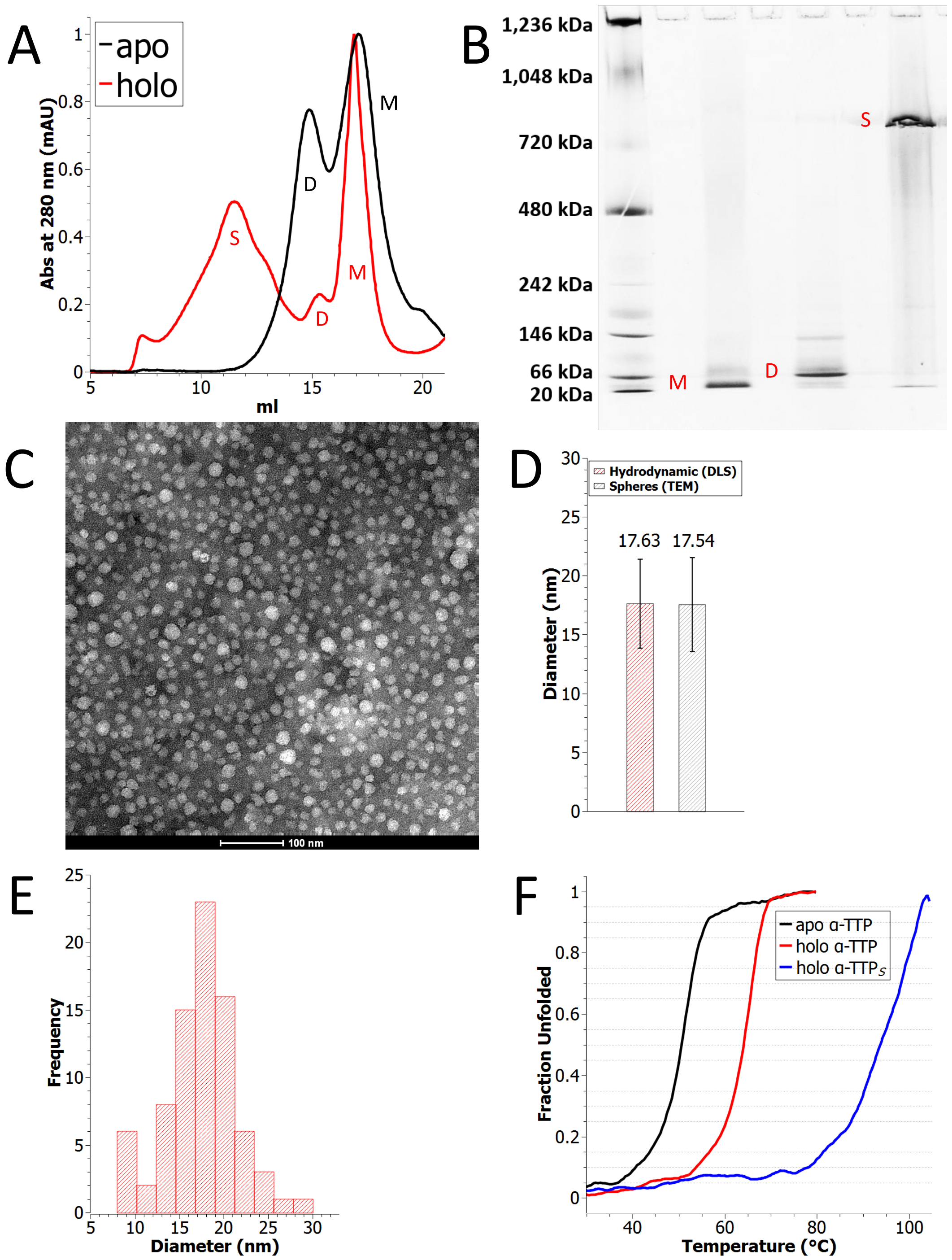

Figure S1: Formation of α-TTP homo-oligomers in presence of α-Tol. (A) SEC traces of preparations of apo-α-TTP (black trace) and α-TTP (red trace). Peak S centres at a retention time that correlates to a mass of 0.76 MDa. (B) Native PAGE of peak fractions M, D and S: Peak S contains an oligomer with an approximate mass of 0.80 MDa, peak D an oligomer with 66 kDa and peak M with 32 kDa corresponding to the monomeric mass of α-TTP. (C) TEM images of the peak fraction of peak S revealed the physical presence of spherical objects. (D) Analysing the TEM pictures with ImageJ indicates an average object diameter of 17.5 ± 4.0 nm, essentially equivalent with the hydrodynamic radius of 17.6 ± 3.8 nm obtained from DLS triplicate measurements. (E) The distribution of the objects from the TEM analysis shows a Gaussian distribution of the diameters centred at 17.0 nm. (F) Thermal denaturation traces monitored by CD spectroscopy at 222 nm for apo-α-TTP, monomeric α-TTP and oligomeric α-TTP$_S$.

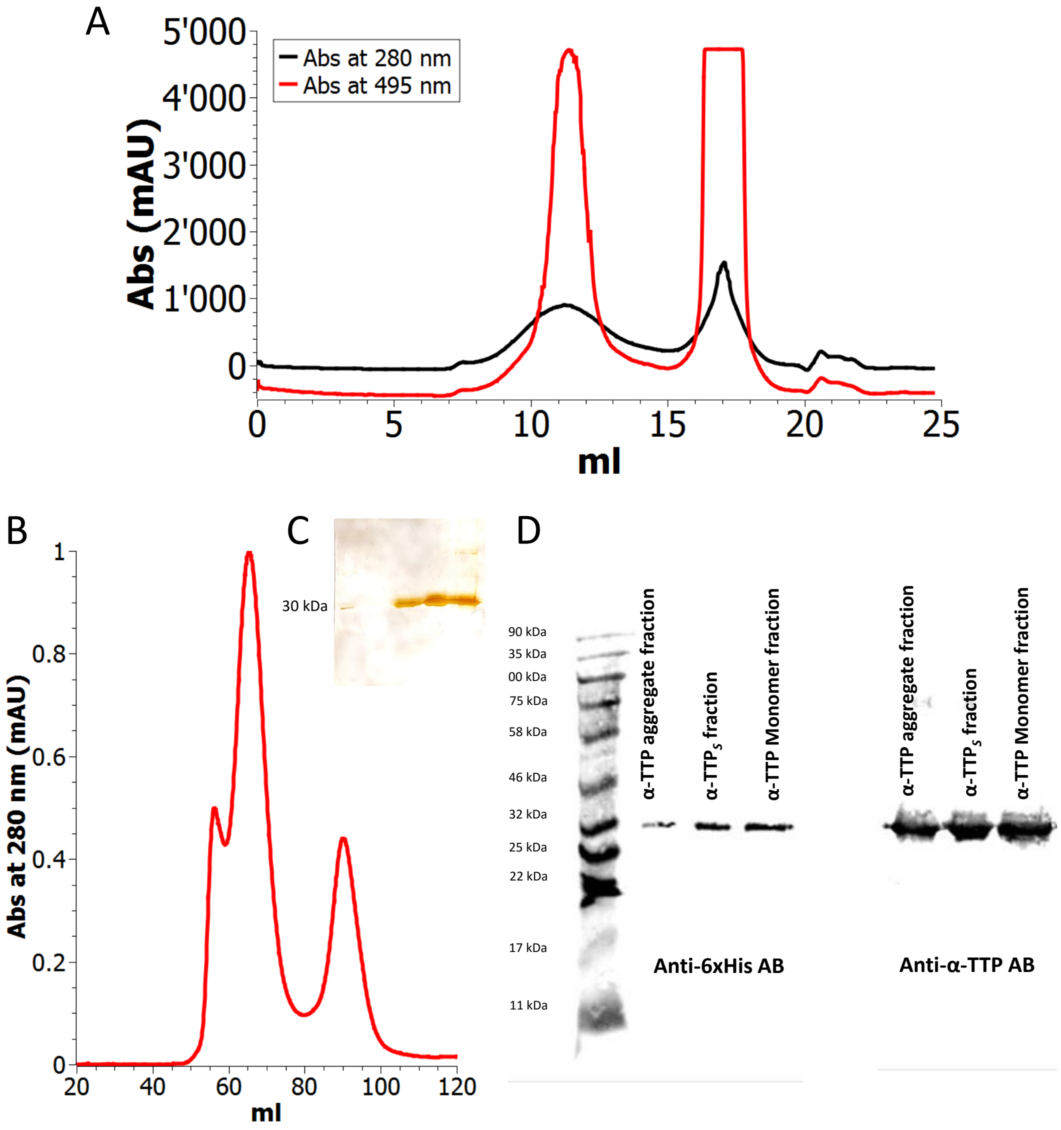

Figure S2: (A) Analytical SEC trace of FITC-labeled *α*-TTP preparations with two major peaks corresponding to FITC-labeled *α*-TTP$_S$ (11.5 ml) and to FITC-labeled *α*-TTP (17.2 ml) (B) Preparative SEC trace of *α*-TTP preparation after loading with *α*-Tol and subsequent dialysis; first peak corresponds to highly aggregated *α*-TTP (≥ 2 MDa), middle peak to *α*-TTP$_S$ and left peak to monomeric *α*-TTP. (C) SDS-PAGE and silver stain of peak fractions from preparative SEC. (D) Western blot of peak fractions from preparative SEC; recombinant *α*-TTP was confirmed by anti-6xHis and by anti-*α*-TTP antibodies.

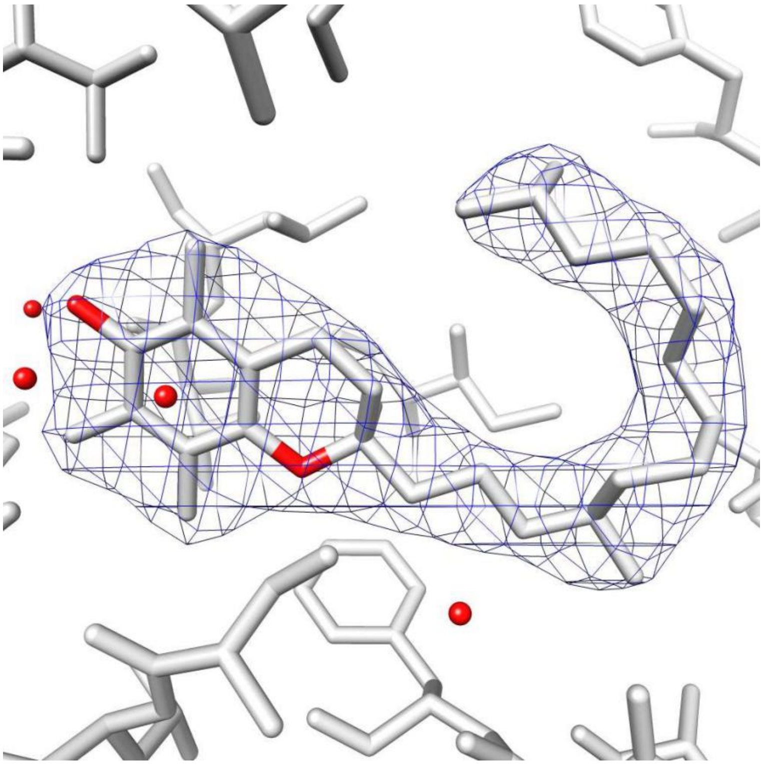

Figure S3: Visualization of *α*-Toc ligand within the protomer; the ligand position is well defined by the X-ray data as shown by the 2Fo-Fc density for it contoured at 1.0 sigma.
